# Human papillomavirus genotyping as a tool for cervical cancer prevention: from commercially available human papillomavirus DNA test to next-generation sequencing

**DOI:** 10.2144/fsoa-2019-0159

**Published:** 2020-07-30

**Authors:** Tauana Christina Dias, Adhemar Longatto-Filho, Nathalia C Campanella

**Affiliations:** 1 Molecular Oncology Research Center, Barretos Cancer Hospital, São Paulo, Brazil; 2 Life & Health Sciences Research Institute (ICVS), School of Health Sciences, University of Minho, Braga, Portugal; 3 ICVS/3B’s – PT Government Associate Laboratory, Braga/Guimarães, Portugal; 4 Laboratory of Medical Investigation (LIM 14), Faculty of Medicine, São Paulo State University, São Paulo, Brazil

**Keywords:** cervical cancer, E6, E7, HPV, immunization, NGS, RT-PCR

## Abstract

The biological importance of human papillomavirus (HPV) in the field of medicine – related to cervical carcinogenesis – has been extensively reported in the last decades. For the first time, a direct correlation between cause and effect to explain a cancer development was completely achieved in medical research. Consequently, the Nobel Prize was awarded to HZ Hausen in 2008 for his efforts to understand the effects of persistent infection of oncogenic types of HPV and malignancy transformation. The aim of the present review was to summarize the principal elements of HPV characteristics and their importance in oncology.

## Lay abstract

It is established that HPV is the main etiologic agent for the development of cervical cancer. With the evolution of diagnosis and molecular biology, many tools have become essential for an early diagnosis and thereby, considerably reducing mortality. Molecular biology continues to advance and provide new perspectives with the use of reverse-transcription PCR in automation and genotyping through next-generation sequencing. This article aims to provide an overview of what is currently used in HPV diagnostic and research and future perspectives with the help of technologies such as next-generation sequencing for screening and vaccination.

Papillomavirus are a large family of virus that infect animals and humans. The most studied and known papillomavirus is the human type. There are more than 150 types of human papillomavirus (HPV) [1–3]. HPV is nonenveloped, with an icosahedral symmetry and a relatively small genome, comprising double-stranded circular DNA containing approximately 8000 base pairs (8 kb) surrounded by 72 capsomeres structures [4].

HPVs are phylogenetically grouped in five different genera: α-, β-, γ-, mu- and nu-HPVs [5]. The alpha group is the largest, containing approximately 65 HPV types, with epithelial cell tropism resulting in infections of the skin and mucous membranes [6]. The HPV alpha type is commonly associated with benign genital warts [6–9]. Around 40 types are associated with the development of cancer, which are further subdivided into low- and high-oncogenic risk [5]. Low-risk types (the most common being HPV11 and HPV6) are most commonly associated with benign genital warts, known clinically as condyloma, but are also implicated in the development of laryngeal papilloma [10]. These condylomas can present size and extension variation but appear as filiform warts. The extension can vary from a few to several centimeters, reaching the mucosa and the skin, commonly presenting on the testis, penis, vulva and anus [10,11].

The high-risk HPVs (HR-HPVs) have been classified as an oncogenic risk by the International Agency for Research on Cancer (Lyon, France), and comprise the types 16, 18, 31, 33, 35, 39, 45, 51, 52, 56, 58, 59, 66 and 68. Within these, type 16, followed by type 18 are the most oncogenic [5]. They are responsible for cancers in the anogenital tract, vagina, vulva, anus, penis and cervix, in addition to some cancers of mouth, pharynx, larynx, esophagus and respiratory tract [11]. Most HPV infections are self-limiting and remain in the individual for an average of 15–18 months until the immune system develops a defense mechanism in response to the pathogen (HPV), which in most cases have asymptomatic presentation in the body [1,12].

HPVs classified in the beta group includes 54 HPV types, which have skin cell tropism and are responsible for most types of benign skin lesions and warts [12]. In some studies, β-HPVs were also correlated to oncogenic potential because they were associated with development of nonmelanoma skin neoplasms, most likely in association with UV radiation [10]. The first known β-HPVs were isolated from skin lesions of patients with a genetic disorder called epidermodysplasia verruciformis [6]. These patients were easily susceptible to infections and completely different when compared with patients with tumors triggered by α-HPV types [13].

The gamma subgroup includes the majority of the known HPVs, with 99 types and even though presenting the same β-HPV biology and infection mechanism, their biological activities are poorly studied and deserve more investigation. They infect human skin and mucosal tissues of the oral cavity and its seroconversion appears to be slow and to increase with age [14,15]. The genera mu and nu include only types 3 and 1 HPV type, respectively [9].

## HPV life cycle

The HPV biological cycle begins as a microtrauma in the mucosa, which allows the virus infection into basal cells of the epithelium. In approximately 3 weeks, it is already possible to detect HPV DNA copies in the basal epithelium as circular DNA, the episomal form. The HPV can develop strategies to avoid immune surveillance, especially the high-risk types [3]. The basal cells are undifferentiated and have low rates of DNA replication, maintaining low HPV DNA copy numbers in the host cell. In this sense, HPV could not be identified in the cytoplasm of the host cells and by using mechanisms to avoid the immune system, as the innate immune system, the virus can survive [3,16–18].

During the basal cell differentiation into epithelial cells, HPV carries many copies of DNA in episomal form in the cell cytoplasm of the cells [13,14]. At this stage, HPV has low replication rates with the same goal as the first, avoiding the immune system. During this period, the immune system is able to identify HPV and develop strategies to eliminate it by the action of TCD4 and TCD8 cells [17,19,20].

The evolution of the virus requires a host to allow DNA replication and viral DNA production [21]. In a second stage, HPV has many copies of DNA in the cytoplasm of cells, resulting in the release of virions that can integrate into the host’s genome, infecting other cells in the other layers of the epithelium, promoting the progression of cancer [19].

## The HPV genome

The HPV genome is divided into three different regions based on functional properties and location. The ‘early’ genes (E) comprise the group of genes *E1, E2, E4, E5, E6* and *E7*, which carry out regulatory functions [3,4]. The ‘late’ genes (L) comprise *L1* and *L2* genes and encode the major and minor capsid proteins, respectively [22]. The L1 region is the most conserved genome region among the HPV types and can reach up to 90% similarity [3,4]. A third region called long control region (LCR) is a noncoding and not well-conserved HPV region, located between the *L1* and *E6* genes, which is involved in controlling the gene expression and replication in the host cells [3,23]. In the HPV genome, early and late regions are identified as the open reading frames and are responsible for the HPV biological cycle, proliferation and replication [24].

In the early region, the *E1* and *E2* genes are responsible for promoting the integration of the HPV genome in human DNA. Through these regions, the HPV has the ability to evolve and progress during the life cycle. E1 binds to E2 forming a dimer of hexamers, when connected, they are able to control viral replication by controlling the host's cellular DNA replication mechanism and inducing the transcription of HPV DNA virus. It is important to draw attention to the fact that the integration of HPV is not always constant, it is necessary that E2 methylation takes place, to found increase of *E6/E7* expression [4,24].

In addition, both E4 and E5 are involved in the early stages of HPV viral replication cycle [19]. Recent studies, using undifferentiated monolayer cell culture systems have demonstrated that both E4 and E5 can activate p38 MAPK, a MAPK important to the activation of caspases and consequently cell death [24].

*E6* and *E7* are the last genes to be activated by stimulating cell growth, inhibition of differentiation and inducing chromosomal instability. The HPV E6 and E7 DNA integration allow the development of a new tumor, which is going to proliferate and invade new tissues [19,25,26]. Because of its central role in cancer we are going to discuss more E6 and E7 in the next section.

The late region, composed by *L1* and *L2* genes, are resposible to produce HPV nonenveloped capsid [24]. HPV infects the epithelium by L1 binding to the basement membrane and by inducing a conformational change in the capsid, exposing L2 to be cleaved, which is a prerequisite to virus uptake and internalization [24,27]. Once the virus has entered the cell, L2 is essential for infection as the endosomal virus and travels toward the nucleus [19,24]. L2 is also important for efficient DNA packaging and virion assembly. When the capsid is formed, HPV is released within the epithelial surface. Free virions can survive in the environment and usually re-infect adjacent cells [24].

## E6 e E7 transformer signaling pathways

After the infection, HPV can integrate into the human genome. The E2 protein loses part of its region and also its function, by controlling *E6* and *E7* genes during HPV genome replication [28]. The *E6* and *E7* oncogenes modulate important signaling pathways – from the host cells cell cycle, to apoptosis and immortalization – allowing viral replication and persistence and becoming the only viral genes that are always retained in cancer cells [29,30].

In the development of cervical cancer (CC), the most important HPV-related human cancer, some genes are downregulated, while others are upregulated by HPV *E6* and *E7* oncogenes, which are able to integrate into human DNA inducing molecular changes promoting the neoplasm development [31].

The protein p53 is one of the main affected by *E6* and *E7* oncogenes. Tumor suppressor p53 is a regulatory gene that is involved in diverse cellular processes such as, apoptosis, DNA repair and cell cycle. The *E6* and *E7* oncogenes are capable of inactivating this gene, allowing tumor development [30]. Specifically, E6 protein binds to p53, becoming activated and recruiting E6AP ubiquitin-protein ligase (E6-associated protein), forming a complex that induces p53 proteasomal degradation [28,32].

Another gene that is also important to HPV carcinogens is *Rb* [30]. The Rb protein (pRB) interacts with E2F and other transcription factors to promote G1-S cell cycle checkpoint control. In HPV-infected cells, E7 has the ability to target pRb for degradation, which is correlated with viral replication and cellular transformation [33].

The *TERT* is another important oncogene. It encodes the catalytic subunit of telomerase enzyme and is crucial to maintenance and regulation of the length of the telomeres [34,35]. In normal somatic cells, telomerase activity is restricted to stem cells, however, recent studies demonstrated that the E6 and E7 proteins from high-risk HPVs are able to induce telomerase reactivation, maintaining the telomere elongation [36]. Telomere maintenance is an important step in tumorigenesis because it confers unlimited proliferative capacity on cancer cells [34,37].

## Cervical cancer

CC is the most important HPV-related human cancer. The association of HPV in CC was first described by HZ Hausen in 1977, for which he was awarded the Nobel Prize in Physiology and Medicine in 2008 [15]. CC is the fourth most diagnosed cancer and fourth most common cancer in women in the world. More than 85% of new cases and more than 87% of the deaths from CC occurred in low income countries [38].

Usually, HR-HPVs cause transient infections that are eventually eliminated over a period of several months by the immune system. If some genetic changes, discussed above, occur in the viral genome and in the infected host cells, a transient infection may be persistent [24]. If not detected and eliminated by the immune system, there is a possibility of progression to cancer [24]. CC is preceded by precursor lesions, a direct result of HPV infection [24]. Precursor lesions, called cervical intraepithelial neoplasia (CIN), are detected by biopsy and classified in three stages [39]. CIN is categorized into CIN1, CIN2 and CIN3, representing the increasing of dysplasia until the establishment of invasive CC [39]. CIN1 is thought to represent a transient cancer HPV infection with a low likelihood of progression to invasive CC and corresponds to low-grade lesions [39]. CIN2 and CIN3, in most cases, represent persistent HPV infection. Approximately 52% of CIN3 is associated with HPV-16 and HPV-18 and represents high-grade lesions [39]. This equates to precancerous disease, but even in CIN3, up to 30% of lesions will regress spontaneously [39].

## Prevention

### HPV vaccination

Vaccination is considered the main frontline program to CC prevention, as primary care [40]. Three types of HPV vaccines are currently available: the bivalent Cervarix^®^, which is a registered trademark of the GlaxoSmithKline (London, UK) group of companies and targets HPV types 16 and 18, which are most common HR-HPV causing CC; quadrivalent, produced by Merck Sharp & Dohme Corp. (NJ, USA) with a commercial name Gardasil^®^, which in addition to preventing HPV 16 and 18, also immunizes against HPV 6 and 11; and more recently, the Gardasil expanded, a nanovalent type, which targets the quadrivalent HPV types, plus the types 31, 33, 45, 52 and 58 [17,18]. Immunization by vaccine programs is certainly the best way to prevent CC. An immunization that covers all oncogenic HPV types, for a total prevention of CC, is the best strategy to prevent the infection of all high oncogenic risk types of virus, however, this requires a big effort from the government and organizations to raise awareness of all the population [41].

### Screening

The secondary prevention strategy for CC is based on screening strategies to detect CIN alterations. The WHO recommends that screening for CC should be performed at least in the target age group (30–60 years), through cytology and visual inspection (VIA) with the use of acetic acid. In women, who test negative on cytology or VIA, the screening interval should be every 3–5 years. In women, who test negative on an HPV test, rescreening should be done after a minimum interval of 5 years [44].

After the long application of pap smear and histology, new technologies to assist in the screening for CC have emerged. Before real-time PCR be applied on a large scale for diagnosis, hybrid capture was a very important tool for HPV screening. It is a technique developed with the advent of molecular biology capable of detecting the presence or absence of HPV virus DNA from low- and high-risk groups using chemo luminescent substrate to produce light, an important step toward the diagnosis with more specificity when compared with conventional cytology [45].

HPV typing becomes an important tool in primary screening for further clinical management. Through available technologies capable of genotyping HPV, it is possible to treat patients from another therapeutic perspective. The challenges are still being overcome, mainly in countries with limited financial resources and health policies [44]. Despite many useful and efficient methodologies currently available for HPV testing [42], Cobas^®^ 4800 platform (Roche Molecular Diagnostics, CA, USA) [43], a system able to genotype HPVs 16 and 18 and 12 other high-risk oncogenic HPV types, was the first FDA approved method to HPV genotyping in clinical settings [18], because of this the method will be briefly described below.

## Real-time PCR technology

The first platform widely used and applied on a large scale for the detection of the HPV virus using real-time PCR technology was Cobas 4800. It has completely autonomous sample preparation with real-time PCR technology, for amplification and detection of HPV, developed by Morris and Nightingale, later commercially explored by Roche [46]. The test simultaneously provides pooled results on high-risk genotypes (HPV 31, 33, 35, 39, 45, 51, 52, 56, 58, 59, 66 and 68) and individual results on the highest risk genotypes, HPV 16 and HPV 18 [47]. Briefly, DNA of the HPV is extracted, purified and prepared for PCR by Cobas × 4800 instruments. The amplification and detection of HR-HPV DNA performed on a Cobas z 4800 analyzer. Both positive and negative control specimens are included in each run, additionally, the test detects β-globin DNA as an internal control of enough cellularity and DNA quality [47]. Cobas 4800 System is a commercially available HPV detection test for use as a first-line primary screening test for CC in women aged 25 and older. Genotyping is only possible at the level of HPV16/18, all other HR-HPVs are detected in a pool and may lead to different clinical conclusions [48].

Additionally, several studies performed on commercially-available HPV DNA detection assays have determined that HPV assays do not detect the same infections in women undergoing primary screening: approximately 30–50% of positive cases show discordance in the specific HPV infection detected. The Validation of HPV Genotyping Tests is an important framework, its function is to validate the HPV assays available on the market, through Validation of HPV Genotyping Tests clinical protocols and trials and be considered safe and valid for the clinic and diagnosis, minimizing the differences from one protocol to another [49].

Therefore, the variation in percentage between one HPV molecular test and another may not only be related to analytical or technical aspects, but also to the viral target sequence selected to detection. According to Galati and collaborators, that studied variations in HPV 16 sequencing in cervical samples, it was possible to observe a significant difference in single nucleotide polymorphisms (SNPs) in the *E6* gene in 74.4% of the samples, when compared with the old sequencing deposited in GeneBank [50]. These findings indicate the need to carefully design the genetic assay to avoid areas with high variability. This may be even more pronounced when testing women from different ethno-geographic origins because specific HPV variants are associated with different patient origins [50].

Modern DNA sequencing methods have enabled complete genomic characterization of cancers on an unprecedented scale. The so-called ‘next-generation sequencing’ (NGS) is enabling the simultaneous analysis of millions of fragments of DNA. The full HPV genotyping is one of the key points of NGS: to detect other genotypes that may increase oncogenic potential in women protected against HPV 16 and 18 infections (through vaccination) [51–53].

It is known that HPV detection assays based on NGS are capable of detecting different variants of oncogenic HPVs. A set of novel sequencing platforms, such as from Illumina (CA, USA) and Thermo Fisher Scientific (MA, USA) completely transformed the scenario, by allowing extensive throughput, while greatly reducing the necessary time and cost of any sequencing endeavor. In the face of increasing migration, these insights will aid identifying high-risk populations for CC screening be more effective. In addition, with NGS assays it is possible to select HPV DNA sequences with maximum sensitivity and specificity for the 14 high-risk genotypes [51–53].

In this scenario, it is possible to compare conventional cytology and HPV testing. Conventional cytology has less of an automated management, requiring a trained professional to analyze samples microscopy, and therefore, classify the lesion of the squamous epithelium according to the smear on a slide. HPV testing has been gaining more space, because it’s easier handling in diagnosis with automation methods and precision in the identification of oncogenic types of HPVs in possible lesions, leading to a faster and more effective process [54].

## Future perspective

New technologies are being used more and more with a greater approach within research. The market follows this evolution and has modernized and developed technologies, such as real-time PCR, making it accessible to anyone.

With the advancement of the application of NGS technology, it is possible to increase the reach and access diverse populations in an effective way, mainly in the early detection of HPV, in which the economic factor is closely linked to prevention. The present study has a perspective that the increasingly advanced technology is a potential ally in prevention, designing the sequencing of all types of high-risk HPV and also in its subtypes. Extrapolating in addition to CC lesions, it is known that persistent HPV infection in other organs and tissues can also develop other types of tumors.

With the advancement of research on HPV genotyping, mainly in the *E6* and *E7* genes, it opens a new perspective for the production of new vaccines for HR-HPV and will become essential in monitoring both vaccinated and unvaccinated women. In addition, an increased of quality in CC screening by the design of potential rapid and efficient molecular tests may improve the detection of CINs and decrease the mortality rates from this type of cancer.

## Conclusion

Different NGS assays applicable to HPV genotyping are under development. This includes the use of different primer design, different NGS platforms and different bioinformatics analysis [53]. In addition, small variations or single nucleotide polymorphisms (SNPs) are easily identified by NGS HPV genotyping methods, which have become useful in epidemiological studies, HPV vaccination surveillance programs, and variants of viral monitors that may escape immunity [43,53].
